# Testing the feasibility of a mobile technology intervention promoting healthy gestational weight gain in pregnant women (txt4two) - study protocol for a randomised controlled trial

**DOI:** 10.1186/s13063-015-0730-1

**Published:** 2015-05-07

**Authors:** Jane Catherine Willcox, Karen Jane Campbell, Elizabeth Anne McCarthy, Shelley Ann Wilkinson, Martha Lappas, Kylie Ball, Brianna Fjeldsoe, Anne Griffiths, Robyn Whittaker, Ralph Maddison, Alexis Shub, Deborah Pidd, Elise Fraser, Nelly Moshonas, David Andrew Crawford

**Affiliations:** Centre for Physical Activity and Nutrition Research, Deakin University, Geelong, Victoria Australia; Department of Obstetrics and Gynaecology, University of Melbourne, Melbourne, Victoria Australia; Mercy Hospital for Women, University of Melbourne, Melbourne, Victoria Australia; Mater Mothers Hospital/Mater Research, Queensland, Australia; Cancer Prevention Research Centre, School of Population Health, University of Queensland, Queensland, Australia; National Institute for Health Innovation, University of Auckland, Auckland, New Zealand

**Keywords:** Pregnancy, Gestational weight gain, Obesity, Overweight, Prenatal care, Cellular phone, mHealth, Text messaging, SMS, Diet, Physical activity

## Abstract

**Background:**

Overweight, obesity and excess gestational weight gain (GWG) are associated with negative health outcomes for mother and child in pregnancy and across the life course. Interventions promoting GWG within guidelines report mixed results. Most are time and cost intensive, which limits scalability. Mobile technologies (mHealth) offer low cost, ready access and individually-tailored support. We aim to test the feasibility of an mHealth intervention promoting healthy nutrition, physical activity and GWG in women who begin pregnancy overweight or obese.

**Methods/Design:**

txt4two is a parallel randomised control trial pilot recruiting women with a singleton, live gestation between 10^+0^ and 17^+6^ weeks at the first hospital antenatal clinic visit. Inclusion criteria are pre-pregnancy BMI > 25 kg/m^2^ and mobile phone ownership. One hundred consenting women will be randomised to intervention or control groups at a 1:1 ratio.

All participants will receive standard antenatal care. In addition, the txt4two intervention will be delivered from baseline to 36 weeks gestation and consists of a tailored suite of theoretically-grounded, evidence-based intervention strategies focusing on healthy nutrition, physical activity and GWG. This includes: mobile phone interactive text messages promoting positive health behaviours, goal setting and self-monitoring; video messages; an information website; and a private moderated Facebook® chat forum.

The primary outcome is the feasibility of the intervention. Secondary outcomes include GWG and participants’ knowledge and behaviour regarding diet and physical activity during pregnancy.

**Discussion:**

Findings will inform the development of larger-scale mHealth programmes to improve the delivery of healthy pregnancy nutrition, physical activity and GWG, that could be widely translated and disseminated.

**Trial registration:**

Australian New Zealand Clinical Trials Registry: ACTRNU111111544397. Date of registration: 19 March 2014.

**Electronic supplementary material:**

The online version of this article (doi:10.1186/s13063-015-0730-1) contains supplementary material, which is available to authorized users.

## Background

Countries around the world have identified obesity prevention as a significant health priority [1]. Interventions timed when populations and individuals are at risk of increasing adiposity can deliver significant quality of life and cost savings, even when the improvement in obesity prevalence is only modest [2]. Evidence suggests that pregnancy is a time of heightened risk for the development of excess adiposity [3]. Promoting healthy weight gain during pregnancy and preventing excess gestational weight gain (GWG) are fast becoming key frontiers in obesity prevention and offer unique opportunities for public health approaches to prevention.

### Excess gestational weight gain

Excess GWG, gaining weight in excess of recommendations during pregnancy [4], is associated with negative health outcomes for maternal and child health in both the short and long term [5]. During pregnancy, excess GWG is associated with increased risk of hypertensive disorders [6], glucose intolerance [7] and negative delivery outcomes [8,9]. It is also predictive of increased infant morbidity and increased foetal growth, including birth weight, large for gestational age and macrosomia [8,9]. Excess GWG is also a predictor of overweight and obesity in women and children in the short, medium and long terms, with evidence of effects up to 21 years post-partum [9-11]. For example, a retrospective cohort study of 10,226 participants showed the odds of overweight in offspring at 7 years increased by 3% for every 1 kg of excess GWG (adjusted odds ratio: 1.03; 95% CI: 1.02/1.05) [10]. This persistent adiposity is suggestive of excess GWG inducing a susceptibility to obesity and potential sequelae, and perpetuating the intergenerational cycle of overweight and obesity.

The prevalence of excess GWG has significantly increased in developed countries over recent decades, with an estimated 35 to 60% of women exceeding the recommended guidelines [6,9,12]. In particular, women who are overweight or obese at conception are at greater risk of exceeding GWG guidelines than those who are not. Pre-pregnancy overweight and obesity amplifies the outcomes related to excess GWG and has been reported to increase the odds of excessive GWG by nearly three-fold [13].

### Gestational weight gain interventions

Systematic reviews of interventions directed at preventing excess GWG demonstrate mixed results [14-17]. A large Cochrane review (27 studies, 3,964 women) that evaluated the effectiveness of interventions for preventing excessive GWG and associated pregnancy complications found insufficient evidence to recommend any intervention for preventing excess GWG due to methodological limitations of included studies and the small observed effect sizes [14]. Conversely, another large systematic review and meta-analysis of ten antenatal dietary and lifestyle intervention randomised controlled trials (RCTs) in obese pregnant women showed an average 2.2 kg reduction in GWG in the intervention compared to the control group participants [18]. This collective evidence base suggests that high-quality trials to evaluate interventions for the promotion of healthy GWG are still needed.

A limitation of the majority of previous interventions has been the heavy reliance on intensive support from clinical providers limiting scalability. The Cochrane review also grouped interventions of varying complexity and intensiveness. They were delivered in clinical maternity or community settings with a prescribed home component utilising either individual or group-based counselling by dietitians, nutritionists or other health workers. The most intensive used intensive counselling and stepwise feedback loops [19]. The least intensive was limited to regular self-weight recording [20].

While evidence for the most effective approaches for preventing excess GWG is limited, there is stronger support for targeting improved nutrition quality, physical activity and knowledge of GWG goals in interventions [21,22]. Inclusion of behaviour change theory in GWG interventions is also limited [23]; however, it is suggested that studies most closely aligned with effective behavioural lifestyle programmes in non-pregnant populations appear most effective in changing targeted health outcomes [24]. Use of health behaviour theories is likely to be important for conceptualising the complexity of behaviour change, in both planning interventions and evaluating outcomes.

### Technology opportunities

As technology becomes more advanced and available, healthcare is utilising technology to deliver improved outcomes [25]. Moreover, the increasing availability of health information in an easily accessible digital format [26], along with the decreased time health providers have in fewer moments of direct patient-provider interaction [25] are changing the health education and information delivery paradigm. Mobile phones have been rapidly and widely adopted among virtually all demographic groups and are increasingly used as a platform for delivering programmes to support the achievement of health objectives, commonly referred to as mHealth [27].

Text messaging or short message service (SMS) is the most widely adopted and one of the least expensive technological features on mobile phones. Text messaging has wide population reach, is relatively low cost, can be individually tailored, does not require technological expertise and allows instant delivery and feedback. As such, text messaging offers potential as a delivery channel for health behaviour interventions [28]. Texting interventions have demonstrated positive impacts on health behaviours, including increased adherence to anti-retroviral therapy and smoking cessation [29]. There is great scope for broader and deeper research into text messaging related to other health behaviours.

Technology-supported dietary and lifestyle interventions in healthy pregnant women are limited [30] and, to the authors’ knowledge, there have been no published studies trialling text message interventions to promote healthy GWG. Given the high prevalence and associated health impost of excess GWG and the high cost of most existing interventions, a new paradigm for healthy GWG promotion is required. The design of an appropriate mHealth intervention to promote healthy GWG building on rigorous scientific development, evaluation, and evidence has the potential to enhance meaningful innovation and best practices. Consistent with recommendations this must be grounded in health behaviour theory, incorporating known mediators for health promotion behaviour, with an adequate sample size to assess feasibility for translation to public health settings [15,23,31].

The mHealth Development and Evaluation framework [32] and others [33] provide guidance in developing new interventions through a staged process. Formative pilot testing of RCTs in the target group is an important first step in developing intervention approaches most likely to be feasible, appealing to, and effective in the target group [34]. Small-scale randomised controlled trials (RCTs) are suitable for feasibility studies of high internal validity when they closely approximate the clinical or community context of a larger scale RCT [34]. Thus, the aim of this study is to test the feasibility of an mHealth intervention to promote healthy nutrition, physical activity and weight gain in pregnant women who are overweight or obese prior to pregnancy.

## Method/Design

### Overview

This protocol describes a two-armed RCT to evaluate the feasibility of an mHealth intervention to promote healthy nutrition, physical activity and weight gain in pregnant women who are overweight or obese prior to pregnancy. The protocol is guided by the Standard Protocol Items: Recommendations for Interventional Trials (SPIRIT), 2013 statement [35] and the Consolidated Standards of Research Trials (CONSORT) - EHEALTH guidelines [36,37]. An additional file shows the SPIRIT checklist (see Additional file 1).

### Ethics approval

Ethics approval was obtained from Deakin University (2014–026) and Mercy Hospital for Women (R13-64) Human Research Ethics Committees.

### Trial entry

Eligible women will be identified at their first hospital antenatal visit to a university affiliated maternity hospital in Melbourne, Australia. Inclusion criteria are women with a singleton, live gestation between 10^+0^ and 17^+6^ weeks who have a self-reported pre-pregnancy, body mass index (BMI) > 25 kg/m^2^ and own a mobile phone. Exclusion criteria include: < 18 years of age; multiple pregnancy; comorbidities requiring significant medical and dietary management; discontinuation of care at hospital; or insufficient English to understand the intervention.

Eligible women will receive an introduction to the study by a researcher, and they will be provided with a plain language statement before obtaining informed consent.

One hundred women will be randomised, following consent, to the intervention or control group in a 1:1 ratio. The randomisation sequence will be obtained using a computer random number generator by JW. Randomisation will occur using numbered cards allocating women to either the intervention or control placed in opaque, sequentially numbered envelopes. Given the nature of the intervention participants will be aware of the group assignment.

### Sample size

The primary outcome of this study is the assessment of feasibility. A sample of 100 participants allows for the estimation of the standard deviation of GWG, a continuous variable, and will give reliable data on the critical recruitment parameters for planning of a larger intervention trial [38]. While the study will not be adequately powered to detect GWG differences between groups, the sample is comparable in size to that of previous GWG studies [39,40] and will provide 80% power with an alpha of 5% to detect a 3-kg difference in GWG (secondary outcome) between the 2 groups, assuming a standard deviation of GWG of 5 kg and allowing for a 10% drop out.

### Standard antenatal care

Participants in this arm will receive standard antenatal care for nutrition, physical activity and weight gain. This consists primarily of information booklets included in the welcome information mailed prior to the first visit to the antenatal clinic and encouragement to weigh at the first visit. This does not include routine provision of diet, physical activity and lifestyle advice although midwives and obstetricians may discuss the topics.

### Intervention

Participants randomised to the intervention will also receive standard care, plus the txt4two intervention focusing on healthy nutrition, physical activity and GWG from baseline to 36 weeks gestation, a common scheduled antenatal appointment.

#### Intervention content

The txt4two intervention content was developed according to evidence-based guidelines. The Institute of Medicine (IOM) GWG guidelines [4] provided the GWG recommendations. The nutrition content is based on the recommendations of the Australian Dietary Guidelines for pregnancy [41] with emphasis on replacement of sugar-sweetened beverages, increased fruit and vegetable intake, reduction of discretionary food groups and consumption of regular meals. The physical activity components are also based on national guidelines for pregnancy [42,43]. The emphasis is on 30 minutes of moderate intensity physical activity on most, if not all, days of the week, reduction of sedentary behaviour and abdominal and pelvic floor strengthening. The behaviour change guidance is informed by the CALO-RE taxonomy of behaviour change techniques [44]. An additional file shows the mapping of intervention content to theoretical constructs (see Additional file 2).

#### Intervention delivery

The multidimensional interventions include tailored text messages, access to a responsive information website, video messages, and chat room interaction via Facebook® (Menlo Park, CA, USA). With the exception of the initial interview and booklet the remainder is accessible on mobile phones and the Internet.

### Initial interview and booklet

At a short initial interview (10 minutes) at recruitment, the trained researcher will outline the programme and provide the participant with a booklet that introduces the texting, website and Facebook® elements as well as short introductions to nutrition, physical activity, GWG and goal setting in pregnancy. In addition, the researcher will direct the participant to appropriate GWG targets for BMI and an individual GWG monitoring tracker [45], encouraging regular weighing and recording. Goal setting will also be emphasised with the participant asked to set a nutrition or physical activity goal to work towards the above-mentioned evidence-based recommendations.

### Text messages

Individually-tailored, interactive text messages will be the core component of the intervention. To standardise the participant texting contact the texting schedule will commence from 16 weeks gestation. Participants will receive four to five texts per week. These texts will deliver information specific to women’s gestational week, encouragement of positive health behaviours and individual behaviour change, monitoring of individual goals and encouragement of self-monitoring of GWG. The text messages were informed by formative work and published data [46] and will be delivered using a two-way text messaging platform, developed by author BF in partnership with a commercial software developer. The package of 121 texts are informed by the CALO-RE taxonomy of behaviour change techniques [44] and delivered in the following categories and frequencies:

1. Gestational progress (weekly)
2. Information and behaviour change direction (twice weekly)
3. Weight self-monitoring (weekly or fortnightly)
4. Weight reporting (monthly)
5. Individual goal checking (weekly or fortnightly)

In keeping with the suggestion that tailoring and personalisation of mHealth programmes encourages behaviour change [31] the text messages are tailored to participant’s gestational week, name and behaviour goal. Text schedules are also tailored to participant’s preferred frequency of self-monitoring and goal checking (for example, weekly or fortnightly) and time of day (for example, early or late morning). Two-way texting is used for the goal checking and weight reporting, which requires participants to respond to the message triggering an automated tailored response from the software. The texting component links participants to the website and Facebook® page.

### Website

A study specific website will outline detailed intervention content information (txt4two website archived by WebCite® at http://www.webcitation.org/6QR3k6uaM. An additional file contains screen shots of the website (see Additional file 3).

Short videos will be embedded in the website. These videos will feature an obstetrician, dietitian or physiotherapist, and outline the benefits of the intervention, explain intervention components, and provide the benefits regarding healthy nutrition.

### Facebook®

Interaction with other participants is encouraged via access to a private Facebook® chat page only accessible by individual invitation. Moderated by a dietitian, participants can pose questions to health professionals and fellow participants as well as report their progress. The dietitian will answer questions within 48 hours and upload tips and information regarding healthy nutrition, physical activity and weight gain at least once per week. Intervention participants are encouraged to join this group during the initial interview with help offered if not proficient with Facebook®.

### Participant incentives

All participants (n = 100) will be provided with a $20 voucher for completing each the initial and the final evaluations. Intervention participants (n = 50) will also be given a $20 iTunes® (Apple Inc., Cupertino, CA, USA) voucher at the initial interview to cover the cost of any text and Internet use.

### Outcome measures

**Figure 1:**
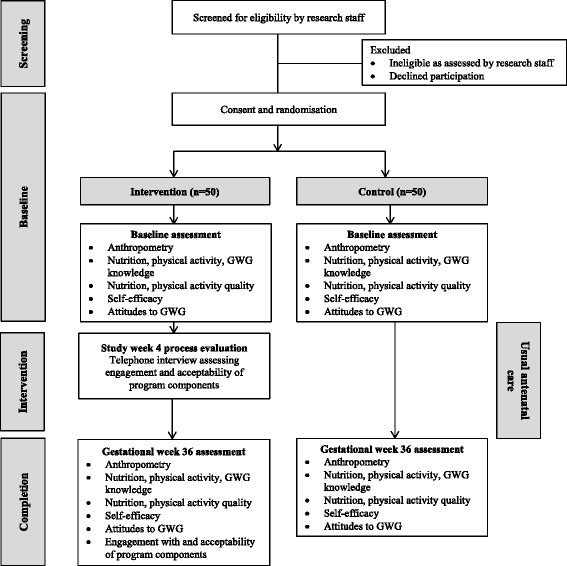
Trial flow diagram.

The primary outcome of intervention feasibility will be measured with programme metrics and participant reported data. The secondary outcomes concerning GWG, nutrition, physical activity and behavioural self-efficacy will assessed with self-reported and anthropometric data at baseline and 36 weeks gestation (Figure 1).

Self-reported participant data will be collected on a computer tablet utilising iSURVEY® (https://www.isurveysoft.com), by the researcher in the antenatal clinic. Additionally, the intervention participants will undertake self-reported process evaluation 4 weeks post texting commencement and at study completion.

#### Primary outcome

The primary outcome of the intervention feasibility will be measured by a composite of programme metrics and participant self-reports. This process evaluation framework is informed by the Process-Evaluation Plan for Assessing Health Programme Implementation [47] and the eCONSORT guidelines [36].

1. *Recruitment and retention*. Proportion of those who are assessed as eligible, those screened and those recruited and randomised. Extent of time taken to recruit. Proportion completing the study and providing evaluation data.
2. *Intervention delivery and fidelity*. Successful intervention delivery to protocol as measure by system reports (delivered text messages, web analytics), and technological difficulties such as downtimes and unexpected events. Contamination of intervention with self-report of other services utilised.
3. *Dose delivered and engagement in the intervention*. Metrics of use of each component: for example, web analytics for page views, time on individual pages, duration of visits, intensity of use, replies to messages, visits to website, views of and contributions to use of Facebook® chat forum. Participant questionnaire will also include participant reported usage of the different components of the intervention.

#### Secondary outcomes

The secondary outcomes will be assessed according to the following measurements:

1. *Gestational weight gain.* GWG will be measured at baseline and 36-week visit will be measured by trained staff with Wedderburn WM301 scales with 0.1 kg accuracy, in light clothing without shoes. Height will be measured on a calibrated stadiometer. Skin fold thickness measurements (biceps, triceps, subscapular) and arm circumference will be measured by trained staff according to a previously published protocol [48].
2. *Nutrition intake*. Quality of diet will be measured by a previously published food frequency questionnaire [49].
3. *Physical activity level*. Physical activity level will be assessed by the Pregnancy Physical Activity Questionnaire (PPAQ) [50]. Physical activity knowledge will be measured with previously published questions [51].
4. *Theoretical behaviour change constructs*:
  - *Knowledge*. GWG, nutrition and physical activity knowledge will be measured by previously utilised questions [51].
  - *Attitude to GWG*. Attitudes toward GWG are measured by the Pregnancy Weight Gain and Attitude Scale [52] with modification.
  - *Self-efficacy*. Self-efficacy for GWG, healthy eating and physical activity will be assessed with measures adapted from a self-efficacy scale for pregnant women [53].
5. *Acceptability of intervention*. Participant questionnaire on acceptability and satisfaction with the intervention. Acceptability of each intervention component and suggestions for improvement will be measured through participant self-report Likert scales.

### Statistical analysis

Analyses will be conducted using Stata (Release 12; StataCorp, College Station, TX, USA). The initial analysis will describe characteristics of participants at baseline. For feasibility assessment categorical variables will be reported as numbers and percentages and continuous variables as mean and standard deviation. Generalised Linear Models [54] will be used to examine intervention effects on the secondary outcomes. Sub-group analysis based on BMI categories will be performed for weight gain per week, and the proportion of participants exceeding IOM guidelines. Results will be analysed by the principle of ‘intention to treat’. The outcome assessors will be blinded to participant allocation.

## Discussion

This paper presents an RCT protocol to determine the feasibility of an mHealth intervention to promote healthy nutrition, physical activity and weight gain in pregnant women who were overweight or obese prior to pregnancy. Previous authors have called for high-quality RCTs promoting healthy GWG grounded in health behaviour theoretical frameworks with adequate sample sizes and feasibility for translation to public health settings [15,23]. If effective, this mHealth intervention offers a programme that could be delivered for large numbers of pregnant women.

Small-scale RCTs that most closely approximate the clinical or community context of a larger-scale RCT help determine whether the intervention should progress to efficacy testing and offer high acceptability to participants and delivery agents, and high internal validity [34]. Given that future intervention success depends on the acceptability of the delivery modality to the target group, and their providers of care, the intervention model and elements of the model require consumer testing to ensure resonance and relevance.

This feasibility study has been designed to provide unique data regarding the suitability of an mHealth-delivered intervention to promote healthy diet, activity and weight in pregnant women. Results of comparisons will help assess relevance, applicability and feasibility of the programme. A potential limitation of the study is the reliance on the self-reported pre-pregnancy weight and nutrition intake and physical activity measures with the potential for recall bias, and hence, biased analyses. This is a common concern for GWG, nutrition and physical activity studies but given the purpose, size and budget of this study, more detailed assessments were not considered feasible. Importantly, the study strengths include the use of multiple technological elements to appeal to a range of preferences and learning styles and the potential for sustainable provision within models of antenatal care.

Findings will inform the development of larger-scale digitally-based programmes to improve the delivery of healthy pregnancy nutrition, physical activity and healthy GWG. The findings of this trial will contribute to the literature on promotion of healthy lifestyles in pregnant women.

## Trial status

The trial has completed recruitment.

## Additional files

Additional file 1:
**SPIRIT 2013 checklist: recommended items to address in a clinical trial protocol and related documents.**
Additional file 2:
**txt4two intervention content mapped to the CALO-RE taxonomy of behaviour change techniques.**
Additional file 3:
**Screen shots of txt4two participant website.**

## Abbreviations

BCT: behaviour change theory
BMI: body mass index
CONSORT: Consolidated Standards of Research Trials
GWG: gestational weight gain
IOM: Institute of Medicine
PPAQ: Physical Activity Questionnaire
RCT: randomised controlled trial
SMS: short message service
SPIRIT: Standard Protocol Items: Recommendations for Interventional Trials
