# Supplementary material for: Testing the feasibility of a mobile technology intervention promoting healthy gestational weight gain in pregnant women (txt4two) - study protocol for a randomised controlled trial
Source: Trials. 2015 May 7;16:209. doi: 10.1186/s13063-015-0730-1 (PMC4426547; doi:10.1186/s13063-015-0730-1)
Supplement: Additional file 2: — txt4two intervention content mapped to the CALO-RE taxonomy of behaviour change techniques. [file 13063_2015_730_MOESM2_ESM.pdf]

Additional file 2: txt4two intervention content mapped to the CALO-RE taxonomy [1] of behaviour change techniques. **Informed by, and adapted from, the model created by Fjeldsoe BS, et all [2].**

Behaviour change technique (listed by taxonomy number)	txt4two intervention component
2. Provide information on consequences of behaviour to the individual	**F2F:** discuss common positive and negative expectations of healthy weight, nutrition and physical activity and provide evidence-based information on realistic expectations<br>**SMS:** targeting technique (e.g. *Jenny. Don't feel guilty for taking time out to exercise, women say they feel more relaxed & happier because they exercise. Jane –txt4two*)<br>**Video:** health professionals outlining positive and negative expectations of healthy weight (obstetrician), nutrition (dietitian) and physical activity (physiotherapist)<br>**Brochure:** provide summary of evidence for positive outcomes of healthy nutrition, physical activity and weight in antenatal period for mother and baby<br>**Website:** provide summary of evidence for positive and negative outcomes of healthy nutrition, physical activity and weight in antenatal period for mother and baby
4. Provide normative information about others' behaviour	**SMS:** targeting technique (e.g. *Want to try a group pregnancy exercise class or swimming? Check out what other women are doing on the facebook group. Jane –txt4two*)<br>**Facebook©:** women share progress, strategies for planning lifestyle change gaols and overcoming barriers<br>**Brochure:** quotes, pictures<br>**Website:** pictures and quotes
5. Goal setting (behaviour)	**F2F:** negotiate initial nutrition/physical activity goal with researcher

	**SMS:** targeting technique (e.g. *Its almost time to plan your SMART goal for txt4two. See the brochure or website for tips. Jane-txt4two*)<br>**Brochure:** outline record weekly goal
6. Goal setting (outcome)	**F2F:** negotiate GWG goal with researcher<br>**Brochure/weight tracker:** view goal trajectory
7. Action planning	**F2F:** discuss plan for first week of nutrition/PA goal (what, when, where, how, with whom?)<br>**Brochure:** plan and record daily sessions (what, when, where, how, with whom?)<br>**SMS:** targeting technique (e.g. *Jenny. You told me you want to eat more vegetables. To achieve this you need to stick to your plan. Check your planner today. Jane-txt4two*)
8. Barrier identification/ problem solving	**F2F:** identify potential barriers and negotiate solutions<br>**SMS:** targeting technique (e.g. *Marie, its OK to miss a day now & then, we all do. The trick is to get back into it ASAP. Review the strategies you planned. Jane-txt4two*)<br>**Facebook©:** women share ideas for overcoming barriers
10. Prompt review of behavioural goals	**SMS:** weekly goal check SMS (e.g. *Hope you are going well Kylie. Did you drink less soft drink this week? Text me back YES or NO so I know. Jane-txt4two*)<br>**Brochure:** planning weekly goals (what, when, where, with whom?)
11. Prompt review of outcome goals	**SMS:** weekly goal check SMS (e.g. *Hope you are going well Karen. Can you please jump on the scales at home or clinic, plot it on your weight tracker and txt me back with the number? Jane- txt4two*)<br>**Brochure/pregnancy weight tracker:** review outcome GWG
13. Provide rewards contingent on successful behaviour	**Brochure:** selection and recording of self-reward based on weekly goal attainment<br>**SMS:** targeting technique if participant replied to weekly goal check (e.g. *Well done Paige! Great start. Make sure you find time for your reward! Jane-txt4two*)

	**Facebook©:** women share ideas for rewards
16. Prompt self-monitoring of behaviour	**SMS:** targeting technique (e.g. *OK Jenny its a new week so lets start fresh. Use your goal planner to remind you of your goal for this week & stick to it. Jane-txt4two*) or for GWG (e.g. *Karen its important to track your weight in pregnancy. Jump on scales @ home today or in clinic & record it on your weight tracker. Jane-txt4two)*<br>**Brochure/pregnancy weight tracker:** track weekly goal attainment and GWG
18.Prompting focus on past successes	**F2F:** discuss previous patterns of eating and physical activity, reasons for stopping<br>**SMS:** targeting technique (e.g. *Quynh take a minute to think about how much better you feel after an exercise session. Remember this next time you don't feel like doing it. Jane-txt4two*)
19. Provide feedback on performance	**F2F:** reviewing objectively measured GWG from baseline assessment<br>**SMS:** targeting technique if participant replied to weekly goal check (e.g. *Fantastic Lynne! It must feel good to prove to yourself you can do it – enjoy that feeling. Jane-txt4two*)
20. Provide information on where and when to perform the behaviour	**SMS:** targeting technique including details of where, when, cost (e.g. *Hi Jenny. Women are enjoying aqua aerobics @ Ivanhoe Aquatic, & Watermarc. Most days and evenings. Costs $12-15. Childcare available. Jane-txt4two*)<br>**Facebook©:** women share information on tips and tricks on implementing lifestyle goals
21. Provide instruction on how to perform the behaviour	**SMS:** targeting technique (e.g. *Carolyn to help with afternoon sweet cravings throw together a mix of unsalted mixed nuts & dried fruit & have a small handful in the afternoon. Jane-txt4two*)<br>**Brochure:** meal plan ideas<br>**Website:** meal plan ideas, recipes

23. Teach to use prompts/cues	**F2F:** discuss positive and negative cues to nutrition/PA change<br>**SMS:** targeting technique (e.g. *Hi Jenny. Always have your exercise clothes clean & ready. Don't let it be an excuse.. Jane-txt4two*) or (e.g. *Hi Shelley. Write a list before you go to the supermarket. Stick to your list and don't get distracted by unhealthy foods at end of aisles & register. Jane-txt4two*)
24. Environmental restructuring	**SMS:** targeting technique (e.g. *Kylie. Cook double size meals & freeze leftovers. Use these extra meals for those nights when you don't have time to cook. Jane-txt4two*)
28. Facilitate social comparison	**SMS:** targeting technique (e.g. *Want to try a group pregnancy exercise class or swimming? Katherine check out what other women are doing in the facebook group. txt4two*)<br>**Facebook© :** women discuss different goals and activities
29. Plan social support/ social change	**SMS:** targeting technique (e.g. *Jenny. Speak to your partner or friend to support you. Make sure they know what your txt4two goal is & what they can do to help you meet it. Jane-txt4two*)<br>**Facebook© :** women from same geographical area discuss goals, strategies and barriers
**Abbreviations**	F2F: initial face-to-face consultation with researcher, GWG: gestational weight gain, PA: physical activity, SMS: short messaging service,

1. Michie S, Ashford S, Sniehotta FF, Dombrowski SU, Bishop A, French DP: A refined taxonomy of behaviour change techniques to help people change their physical activity and healthy eating behaviours: The CALO-RE taxonomy. *Psychol Health* 2011, 26(11):1479-1498.
2. Fjeldsoe BS, Miller YD, O'Brien JL, Marshall AL: Iterative development of MobileMums: a physical activity intervention for women with young children. *International Journal of Behavioral Nutrition and Physical Activity* 2012, 9(151).
