# Supplementary figures and images for: Testing the feasibility of a mobile technology intervention promoting healthy gestational weight gain in pregnant women (txt4two) - study protocol for a randomised controlled trial
Source: Trials. 2015 May 7;16:209. doi: 10.1186/s13063-015-0730-1 (PMC4426547; doi:10.1186/s13063-015-0730-1)

## Additional file 3: Screen shots of txt4two participant website

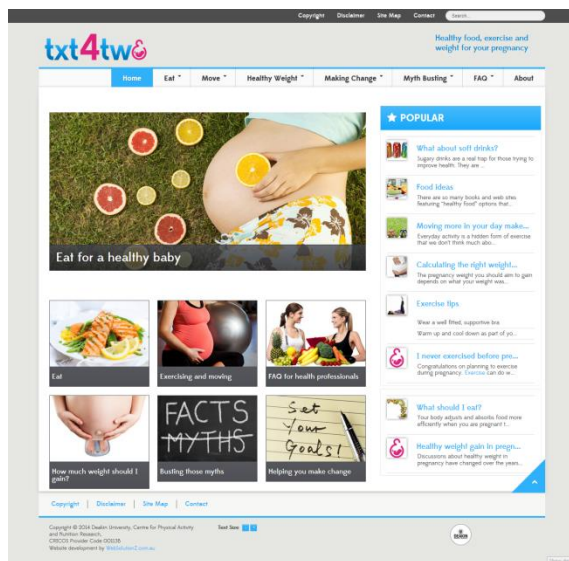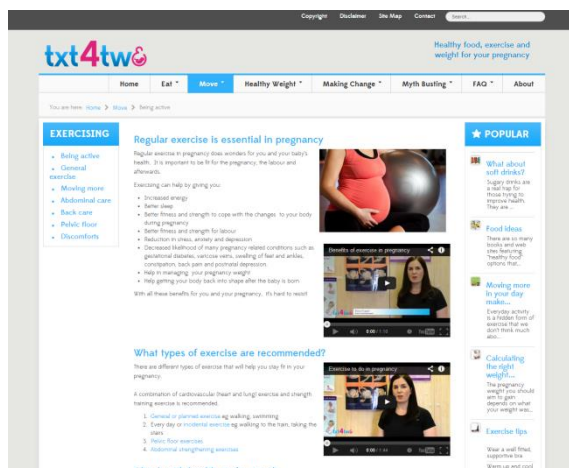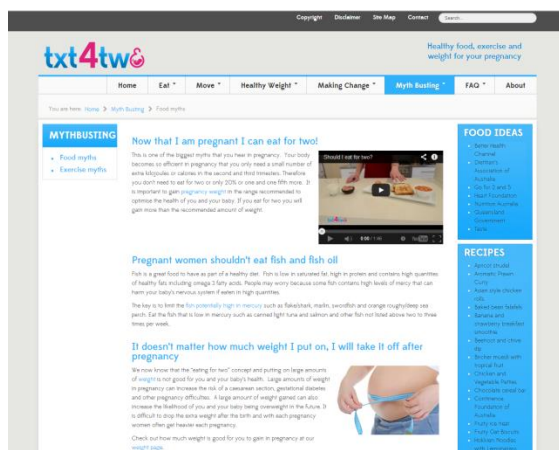

Supplement: Additional file 3: — Screen shots of txt4two participant website. [file 13063_2015_730_MOESM3_ESM.pdf]
